# Association between maternal occupational exposure to cleaning chemicals during pregnancy and childhood wheeze and asthma

**DOI:** 10.3389/fepid.2023.1166174

**Published:** 2023-04-19

**Authors:** Melissa A. Herrin, Allison R. Sherris, Logan C. Dearborn, Christine T. Loftus, Adam A. Szpiro, Paul E. Moore, Margaret A. Adgent, Emily S. Barrett, Ruby H. N. Nguyen, Kecia N. Carroll, Catherine J. Karr

**Affiliations:** ^1^ Department of Medicine, School of Medicine, University of Washington, Seattle, WA, United States; ^2^ Department of Environmental and Occupational Health Sciences, School of Public Health, University of Washington, Seattle, WA, United States; ^3^ Department of Biostatistics, School of Public Health, University of Washington, Seattle, WA, United States; ^4^ Division of Pediatric Allergy, Immunology, and Pulmonary Medicine, Department of Pediatrics, Vanderbilt University Medical Center, Nashville, TN, United States; ^5^ Department of Health Policy, Vanderbilt University Medical Center, Nashville, TN, United States; ^6^ Department of Biostatistics and Epidemiology, School of Public Health, Rutgers, The State University of New Jersey, Piscataway, NJ, United States; ^7^ Environmental and Occupational Health Sciences Institute, Rutgers, The State University of New Jersey, Piscataway, NJ, United States; ^8^ Division of Epidemiology and Community Health, School of Public Health, University of Minnesota, Minneapolis, MN, United States; ^9^ Department of Environmental Medicine and Public Health, Icahn School of Medicine at Mount Sinai, New York, NY, United States; ^10^ Department of Pediatrics, Icahn School of Medicine at Mount Sinai, New York, NY, United States; ^11^ Department of Pediatrics, School of Medicine, University of Washington, Seattle, WA, United States; ^12^ Department of Epidemiology, School of Public Health, University of Washington, Seattle, WA, United States

**Keywords:** cleaning chemicals, childhood wheeze, childhood asthma, occupational exposure, prenatal exposure, environmental exposure, respiratory outcomes

## Abstract

**Background:**

Asthma is a leading cause of childhood morbidity in the U.S. and a significant public health concern. The prenatal period is a critical window during which environmental influences, including maternal occupational exposures, can shape child respiratory health. Cleaning chemicals are commonly encountered in occupational settings, yet few studies have examined the potential link between prenatal occupational exposures to cleaning chemicals and risk of childhood wheeze and asthma.

**Methods:**

We evaluated the potential influence of maternal occupational exposure to cleaning chemicals during pregnancy on pediatric asthma and wheeze at child age 4–6 years in 453 mother-child pairs from two longitudinal pregnancy cohorts, TIDES and GAPPS, part of the ECHO prenatal and early childhood pathways to health (ECHO-PATHWAYS) consortium. Maternal occupational exposure to cleaning chemicals was defined based on reported occupation and frequency of occupational use of chemicals during pregnancy. Child current wheeze and asthma outcomes were defined by parental responses to a widely-used, standardized respiratory outcomes questionnaire administered at child age 4–6 years. Multivariable Poisson regression with robust standard errors was used to estimate relative risk (RR) of asthma in models adjusted for confounding. Effect modification by child sex was assessed using product interaction terms.

**Results:**

Overall, 116 mothers (25.6%) reported occupational exposure to cleaning chemicals during pregnancy, 11.7% of children had current wheeze, and 10.2% had current asthma. We did not identify associations between prenatal exposure to cleaning chemicals and current wheeze [RR_adjusted_ 1.03, 95% confidence interval (CI): 0.56, 1.90] or current asthma (RR_adjusted_ 0.89, CI: 0.46, 1.74) in the overall sample. Analyses of effect modification suggested an adverse association among females for current wheeze (RR 1.82, CI: 0.76, 4.37), compared to males (RR 0.68, CI: 0.29, 1.58), though the interaction *p*-value was >0.05.

**Conclusion:**

We did not observe evidence of associations between maternal prenatal occupational exposure to cleaning chemicals and childhood wheeze or asthma in the multi-site ECHO-PATHWAYS consortium. We leveraged longitudinal U.S. pregnancy cohorts with rich data characterization to expand on limited and mixed literature. Ongoing research is needed to more precisely characterize maternal occupational chemical exposures and impacts on child health in larger studies.

## 1. Introduction

Childhood asthma affects approximately 8% of children in the U.S. and represents a significant public health concern (1). Asthma is a complex, chronic inflammatory respiratory disease, characterized by airway hyperresponsiveness, inflammation, and obstruction, and is often triggered by environmental factors (2). Symptoms include episodes of breathlessness, coughing, and wheezing, and asthma that develops in childhood has a profound impact on lifelong lung health, including airway remodeling and increased risk for adult asthma (3, 4). The prenatal period is a significant window during which interactions between genetics and environmental exposures, including environmental toxicants, modulate fetal lung development and immunologic responses that influence the risk, incidence, and severity of allergic diseases and asthma (3, 5–8).

Cleaning and disinfectant products are complex mixtures of chemicals, including irritants and potential sensitizers (9–11). Prior investigations have found robust, consistent epidemiological evidence that both home and occupational exposures to chemicals involved in cleaning and janitorial tasks, including disinfectants, fragrances, and solvents, increase risk for respiratory symptoms and asthma in adults (9, 11–18). In children, evidence also suggests a link between use of cleaning chemicals and sprays in the home with airway inflammation, persistent wheeze, lung function abnormalities, and increased risk of asthma (19–21).

By contrast, maternal environmental exposures during pregnancy and preconception, including occupational exposures to cleaning chemicals, have been found to be associated with childhood wheeze and asthma, though mechanisms are not yet fully understood (3, 10, 22–24). Several parental occupations are associated with higher risk of childhood respiratory outcomes, including jobs involving cleaning and chemical disinfection (22, 23, 25). Thus far, few studies have examined the potential link between maternal occupational exposures to cleaning agents specifically in the prenatal period and childhood asthma. A recent European cohort study found that both asthma and a related atopic condition in childhood (e.g., allergic rhinitis) were linked to prenatal exposure to cleaning agents (10). Finally, pre-adolescent boys have an increased prevalence of asthma, and child sex has been found to modify the relationship between prenatal environmental exposures and child airway outcomes (26–28), but few studies have specifically focused on the modifying role of child sex in prenatal occupational exposure to cleaning chemicals (22).

We contribute to this limited evidence base by evaluating the potential influence of maternal occupational exposure to cleaning chemicals during the prenatal period on pediatric asthma and wheeze outcomes at child age 4–6 years. Furthermore, we evaluate whether there is evidence of sex-specific associations. We utilize asthma and wheeze data collected in middle childhood in the ECHO prenatal and early childhood pathways to health consortium (ECHO-PATHWAYS), a multi-site longitudinal study combining three U.S. pregnancy cohorts with extensive pregnancy exposure, child outcome and covariate characterization. We hypothesize that maternal exposure to cleaning chemicals during pregnancy will be associated with increased risk of asthma and wheeze at age 4–6 years and that effects will vary by child sex.

## 2. Materials and methods

### 2.1. Study setting and population

The study participants were mother-child pairs from two ECHO-PATHWAYS consortium pregnancy cohorts: the Global Alliance to Prevent Prematurity and Stillbirth (GAPPS) and The Infant Development and Environment Study (TIDES) (29).

GAPPS participants were enrolled between 2011 and 2014 from three hospitals in Seattle, WA and Yakima, WA. Inclusion criteria included being 18 years or older, able to speak and write English, and planning to deliver at the study hospital in which they were enrolled. Eligible mother-child dyads were recruited into ECHO-PATHWAYS when the children were 4–6 years old and attended clinic visits at age 4–6 years and age 8–9 years. TIDES participants were recruited during the first trimester of pregnancy from participating academic medical centers, from 2010 to 2012: San Francisco, CA; Minneapolis, MN; Rochester, NY; and Seattle, WA. Women were eligible if they were 18 years old or older, planning to deliver at one of the participating study hospitals, and having a low-risk singleton pregnancy at enrollment. Mother-child pairs were administered questionnaires and/or attended clinic visits at ages 4–5 years, 6 years and 7 years (30, 31). This analysis includes participants who completed both the occupational exposure questionnaire and the International Study of Asthma and Allergies in Childhood (ISAAC) questionnaire (32, 33) at child age 4–6 years. Because visits varied in composition and not all surveys were administered at all visits, only a subset of the participants who attended visits have both exposure and outcome data required for the main study question and are included in these analyses.

Participants provided informed consent. Data were analyzed by the University of Washington (UW) ECHO-PATHWAYS team and study protocols were approved by the UW Institutional Review Board (IRB).

### 2.2. Occupational prenatal exposure to cleaning chemicals

Our primary exposure of interest, maternal occupational exposure to cleaning chemicals during the prenatal period, was assessed using questionnaires administered to primary caregivers regarding job titles, occupational activities, and exposures during the prenatal period. Questionnaires were administered at the GAPPS child age 4–6 visit (GAPPS 4–6), GAPPS child age 8–9 visit (GAPPS 8–9) or TIDES child age 7 visit (TIDES 7). In both GAPPS and TIDES, prenatal exposure to cleaning chemicals was defined as meeting any of the following: (1) answered “Yes” to “*Did the biological mother work in any of the following industries during pregnancy: Janitor or house cleaner?*”; (2) answered “Yes” to “*Did the biological mother do any of the following activities at her job during pregnancy: Clean floors, sinks, or toilets?”;* (3) answered “Some days” or “Every day” to “*How often did the biological mother use janitorial chemicals or cleaners at her job during pregnancy?”.*

### 2.3. Child airway outcomes

We defined our primary outcomes as current wheeze and current asthma and our secondary outcome as strict asthma as reported between child ages 4–6 using the ISAAC questionnaire. The categorization of outcomes is similar to that used previously in ECHO-PATHWAYS consortium research (27–29, 34, 35). Current wheeze was defined as a positive response to “*Has the child had wheezing or whistling in the chest in the past 12 months?”* Current asthma was defined as positive responses to two of the following: current wheeze, ever asthma (defined as positive response to the question “*Has your child ever had asthma?”*), and asthma medication use (*“Does the child use any medications for treatment of recurrent cough, recurrent wheezing or asthma?”*). Strict asthma was defined as positive response to the question “*Has your child ever had asthma?”* as well as either current wheeze or asthma medication use (35, 36).

### 2.4. Statistical analysis

Demographic and behavioral characteristics of mother-child pairs were summarized overall and by cohort.

Modified multivariable Poisson regression with robust standard errors was used to estimate associations [adjusted risk ratios (RR)] between exposure and outcomes. The primary analyses investigated the association between prenatal exposure to cleaning chemicals (yes/no) and primary outcomes (current wheeze and current asthma) using separate models for each outcome. Secondary analyses investigated the association between prenatal exposure to cleaning chemicals and strict asthma.

We used a staged modeling approach for covariate adjustment by fitting minimally adjusted, fully adjusted (main model), and extended models. Covariates were selected *a priori* based on a literature search to identify asthma and wheeze risk factors that may be correlated with the exposure and included maternal, child, and household demographic, health, and socioeconomic factors. Minimally adjusted models were adjusted for child age, child sex, and study site. Main models further adjusted for self-identified maternal race (White, Asian, or other) and maternal ethnicity (Hispanic/Latino or non-Hispanic/Latino), education at enrollment (less than high school, high school completion, graduated college/technical school, or any graduate school/professional), maternal history of asthma (yes/no), maternal age at delivery (years, continuous), maternal self-report of smoking status at enrollment (yes/no), household size category (<4, 4, 5, >5), region-and inflation-adjusted household income (continuous, $USD), postnatal second-hand smoke exposure (yes/no), season of birth (warm [April through September]/cold [October through March]), and firstborn status (yes/no). Extended models additionally adjusted for two potential confounders that may also act as mediators: preterm birth at less than 37 weeks (binary) and birthweight (continuous). To evaluate whether the association between prenatal exposure to cleaning chemicals and childhood asthma is modified by child sex, we tested for a statistical interaction using multiplicative interaction terms. The primary models, effect modification analysis, and sensitivity analyses utilized multiple imputation by chained equations (MICE) to impute missing covariates (37).

We conducted multiple sensitivity analyses to assess the robustness of findings to modeling approach. In all cases, the sensitivity analyses were compared to the main model. Demographic and behavioral characteristics were also summarized for participants included in this study and those who were excluded but still attended the age 4–6 visit. To explore whether results were influenced by site- and cohort- specific associations, leave-one-out analyses were conducted in which the main analysis was repeated with one cohort or site removed in each iteration. To assess whether bias was introduced due to variation in ability to recall exposures at different child ages, we additionally adjusted for visit of exposure questionnaire completion [age 4–6 or 7/8–9 visit (binary)]. We performed additional sensitivity analyses in which we adjusted for urinary cotinine (continuous) as a marker of maternal smoking and exposure to environmental tobacco smoke during pregnancy (38), measured during the second trimester visit, and whether the child had ever been diagnosed with bronchiolitis (yes/no). Maternal pregnancy tobacco smoke exposure and early childhood bronchiolitis are both associated with development of childhood asthma (1, 39); however, we did not include these in the main models because they were not collected for the GAPPS participants who completed the exposure recall at age 4–6 (*N* = 96) per study protocols. To more precisely capture clinically relevant exposure to cleaning chemicals, prenatal cleaning practices and prenatal cleaning frequency were assessed independently. Separate analyses were performed defining prenatal exposure as either (1) answered “Yes” to “*Did the biological mother do any of the following activities at her job during pregnancy—Clean floors, sinks, or toilets?”* or (2) answered “Some days” or “Every day” to “*How often did the biological mother use janitorial chemicals or cleaners at her job during pregnancy?”* Finally, we repeated the primary analysis using complete cases only.

All analyses were conducted in R version 4.2.2 and significance was assessed at an *α* level of 0.05.

## 3. Results

Overall, 453 pregnancy exposure and occupation recall questionnaires were completed, 239 in GAPPS and 214 in TIDES (Table 1). Of these, 116 mothers (25.6%) were classified as having been exposed to cleaning chemicals at their job during pregnancy. There was overlap among classification of exposure: ten mothers (2.2%) worked as a janitor or house cleaner, 77 (17%) cleaned floors, sinks, or toilets as part of their job, and 88 (19.4%) used janitorial chemicals or cleaners in their job some days or every day (Table 2). Mean child age at outcome assessment was 5.8 years [standard deviation (SD) 0.7] with an interquartile range (IQR) of 5.3–6.2 (Table 1). The child participants were 54.3% male and 45.7% female. The overall prevalences of current wheeze, current asthma, and strict asthma were 11.7% (*N* = 53), 10.8% (*N* = 49), and 7.7% (*N* = 35), respectively (Table 3).

**Table 1:** Descriptive characteristics of the study population by cohort.

	^ ^	Cohort^a^	
	Total^b^	TIDES	GAPPS
	(*N* = 453)	(*N* = 214)	(*N* = 239)
Maternal race			
White	346 (76.4%)	187 (87.4%)	159 (66.5%)
Black	20 (4.4%)	0 (0%)	20 (8.4%)
Asian	24 (5.3%)	15 (7.0%)	9 (3.8%)
Native Hawaiian/Other Pacific Islander	1 (0.2%)	0 (0%)	1 (0.4%)
American Indian/Alaska Native	2 (0.4%)	1 (0.5%)	1 (0.4%)
Other	26 (5.7%)	17 (7.9%)	9 (3.8%)
Multiple race	16 (3.5%)	1 (0.5%)	15 (6.3%)
Missing	18 (4.0%)	18 (8.4%)	0 (0%)
Maternal ethnicity			
Hispanic or Latino	38 (8.4%)	17 (7.9%)	21 (8.8%)
Not Hispanic or Latino	401 (88.5%)	197 (92.1%)	204 (85.4%)
Missing	14 (3.1%)	0 (0%)	14 (5.9%)
Maternal education			
Less than high school	14 (3.1%)	11 (5.1%)	3 (1.3%)
High school	79 (17.4%)	28 (13.1%)	51 (21.3%)
College/technical school	170 (37.5%)	65 (30.4%)	105 (43.9%)
Graduate or Professional degree	190 (41.9%)	110 (51.4%)	80 (33.5%)
Maternal history of asthma			
Yes	60 (13.2%)	24 (11.2%)	36 (15.1%)
No	365 (80.6%)	177 (82.7%)	188 (78.7%)
Missing	28 (6.2%)	13 (6.1%)	15 (6.3%)
Maternal Delivery Age (years)			
Mean (SD)	32.1 (5.3)	31.8 (5.4)	32.3 (5.1)
Median (IQR)	32 (29–36)	32 (28–36)	32 (29–36)
Missing	29 (6.4%)	0 (0%)	29 (12.1%)
Child sex			
Male	246 (54.3)	110 (51.4%)	136 (56.9%)
Female	207 (45.7%)	104 (48.6%)	103 (43.1%)
Preterm birth			
Yes	67 (14.8%)	13 (6.1%)	54 (22.6%)
No	364 (80.4%)	198 (92.5%)	166 (69.5%)
Missing	22 (4.9%)	3 (1.4%)	19 (7.9%)
Season of birth			
Warm	237 (52.3%)	109 (50.9%)	126 (52.7%)
Cold	216 (47.7%)	105 (49.1%)	113 (47.3%)
Birthweight (grams)			
Mean (SD)	3,252 (705.6)	3,387 (500.8)	3,122 (839)
Median (IQR)	3,316 (2940–3710)	3,358 (3071–3700)	3,274 (2755–3716)
Missing	120 (26.5%)	15 (7.0%)	22 (9.2%)
Firstborn status			
Yes	130 (28.7%)	35 (16.4%)	90 (37.7%)
No	305 (67.3%)	172 (80.4%)	138 (57.7%)
Missing	18 (4.0%)	7 (3.3%)	11 (4.6%)
Ever bronchiolitis^c^			
Yes	31 (6.8%)	14 (6.5%)	17 (7.1%)
No	302 (66.7%)	185 (86.4%)	117 (49.0%)
Missing	120 (26.5%)	15 (7.0%)	105 (43.9%)
Child age at 4-6 visit (years)			
Mean (SD)	5.8 (0.7)	6.2 (0.4)	5.5 (0.7)
Median (IQR)	6 (5.3–6.2)	6.1 (6.0–6.2)	5.4 (5.1–5.9)
Missing	18 (4.0%)	7 (3.3%)	11 (4.6%)
Household size			
<4	80 (17.7%)	40 (18.7%)	40 (16.7%)
4	204 (45.0%)	101 (47.2%)	103 (43.1%)
5	83 (18.3%)	35 (16.4%)	48 (20.1%)
>5	50 (11.0%)	19 (8.9%)	31 (13.0%)
Missing	36 (7.9%)	19 (8.9%)	17 (7.1%)
Adjusted income ($USD)			
Mean (SD)	$114,004 ($56,745)	$117,237 ($59,070)	$111,188 ($54,193)
Median (IQR)	$110,813 ($67,648–$172,511)	$110,813 ($67,676–$172,511)	$105,682 ($67,648–$147, 955)
Missing	32 (7.1%)	18 (8.4%)	14 (5.9%)
Smoking self-report			
Yes	15 (3.3)	10 (4.7%)	5 (2.1%)
No	436 (96.2%)	104 (48.6%)	232 (97.1%)
Missing	2 (0.4%)	0 (0%)	2 (0.8%)
Averaged cotinine^d^			
Mean (SD)	29.7 (169.2)	40.7 (206.1)	11.4 (79.2)
Median (IQR)	0.01 (0.01–0.06)	0.01 (0.01–0.07)	0.01 (0.001–0.02)
Missing	113 (24.9%)	2 (0.9)	111 (46.4%)
Postnatal second-hand smoke exposure			
Yes	127 (28.0%)	121 (56.5%)	16 (6.7%)
No	300 (66.2%)	93 (43.5%)	207 (86.6%)
Missing	16 (3.5%)	9 (4.2%)	16 (6.7%)
Cohort Site			
GAPPS			
Seattle, WA	164 (36.2%)		164 (68.6%)
Yakima, WA	75 (16.6%)		75 (31.4%)
TIDES			
Minneapolis, MN	56 (12.4%)	56 (26.2%)	
Rochester, NY	49 (10.8%)	49 (22.9%)	
San Francisco, CA	63 (12.9%)	63 (29.4%)	
Seattle, WA	46 (10.2%)	46 (21.5%)	

^a^ Percentages are within cohort.

^b^ Percentages are within total group.

^c^ Bronchiolitis history was not surveyed for the subset of GAPPS participants who completed the recall survey at age 4–6. Bronchiolitis was unavailable for *N* = 11 from the GAPPS age 8–9 recall group.

^d^ Measured in nanograms per milliliter (ng/mL). Cotinine was not measured for the subset of GAPPS participants who completed the recall survey at age 4–6. Cotinine was unavailable for *N* = 15 from the GAPPS age 8–9 recall group.

**Table 2:** Occupational exposure to cleaning chemicals^a^ among pregnant individuals in the study population.

		Cohort	
	Total	TIDES	GAPPS
	(*N* = 453)	(*N* = 214)	(*N* = 239)
Exposure (composite, prenatal)			
Yes	116 (25.6%)	48 (22.4%)	68 (28.5%)
No	337 (74.4%)	166 (77.6%)	171 (71.5%)
Works as janitor or house cleaner			
Yes	10 (2.2%)	5 (2.3%)	5 (2.1%)
No	442 (97.6%)	209 (97.7%)	233 (97.5%)
Missing	1 (0.2%)	0 (0%)	1 (0.4%)
Cleans floors, sinks, or toilets at job			
Yes	77 (17%)	40 (18.7%)	37 (15.5%)
No	375 (82.8%)	173 (80.8%)	202 (84.5%)
Missing	1 (0.2%)	1 (0.5%)	0 (0%)
Use janitorial chemicals or cleaners at job			
Some days	72 (15.9%)	22 (10.3%)	50 (20.9%)
Every day	16 (3.5%)	6 (2.8%)	10 (4.2%)
Never	362 (79.9%)	183 (85.5%)	179 (74.9%)
Missing	3 (0.7%)	3 (1.4%)	0 (0%)

^a^ Maternal occupational exposure to cleaning chemicals during the prenatal period was assessed using questionnaires administered to primary caregivers and were completed at the GAPPS child age 4–6 visit, GAPPS child age 8–9 visit or TIDES child age 7 visit. Exposure was defined as meeting any of the following: (1) answered of “Yes” to “Did the biological mother work in any of the following industries during pregnancy: Janitor or house cleaner?”; (2) answered “Yes” to “Did the biological mother do any of the following activities at her job during pregnancy: Clean floors, sinks, or toilets?”; (3) answered “Some days” or “Every day” to “How often did the biological mother use janitorial chemicals or cleaners at her job during pregnancy?”.

**Table 3:** Asthma and wheeze outcomes at age 4–6 years in the study population.

		Cohort	
	Total	TIDES	GAPPS
	(*N* = 453)	(*N* = 214)	(*N* = 239)
Current wheeze			
Yes	53 (11.7%)	33 (15.4%)	20 (8.4%)
No	373 (82.3%)	174 (81.3%)	199 (83.3%)
Missing	27 (6.0%)	7 (3.3%)	20 (8.4%)
Current asthma			
Yes	49 (10.2%)	13 (6.1%)	36 (15.1%)
No	389 (85.9%)	183 (85.5%)	206 (86.2%)
Missing	15 (3.3%)	7 (3.3%)	8 (3.3%)
Strict asthma			
Yes	35 (7.7%)	14 (6.5%)	20 (8.4%)
No	392 (86.5%)	193 (90.2%)	199 (83.3%)
Missing	27 (5.8%)	7 (3.3%)	20 (8.4%)

In our primary analysis, we did not observe associations between the composite measure of prenatal cleaning chemical exposure and current wheeze in the main model (RR 1.03, CI: 0.56, 1.90) or current asthma (RR 0.89, CI: 0.46, 1.74) (Table 4). Results were similar in the minimally adjusted and extended models. Similarly, our secondary analysis found no association between prenatal exposure to cleaning chemicals and strict asthma (RR 0.82, CI: 0.33, 2.02) (Table 4).

**Table 4:** Association between maternal occupation exposure to cleaning chemicals during pregnancy and airway outcomes.

	Minimally-adjusted model^a^		Main model^b^		Extended model^c^	
Primary outcomes	Adjusted RR (95% CI)	*p*-value	Adjusted RR (95% CI)	*p*-value	Adjusted RR (95% CI)	*p*-value
Current wheeze	0.90 (0.49–1.64)	0.72	1.03 (0.56–1.90)	0.92	1.03 (0.55–1.90)	0.65
Current asthma	0.86 (0.45–1.64)	0.65	0.89 (0.46–1.74)	0.74	0.88 (0.45–1.71)	0.74
Secondary outcome						
Strict asthma	0.70 (0.31) – 1.55	0.38	0.82 (0.33–2.02)	0.67	0.80 (0.33–1.96)	0.63

^a^ Minimally adjusted models were adjusted for child age, child sex, and study site.

^b^ Main models were further adjusted for maternal race, maternal ethnicity, education at enrollment, maternal history of asthma, maternal age at delivery, and maternal self-report of smoking status at enrollment, household size category, regional-and inflation-adjusted household income, postnatal second-hand smoke exposure, season of birth, and firstborn status.

^c^ Extended models were additionally adjusted for preterm birth and birthweight.

**Figure 1:**
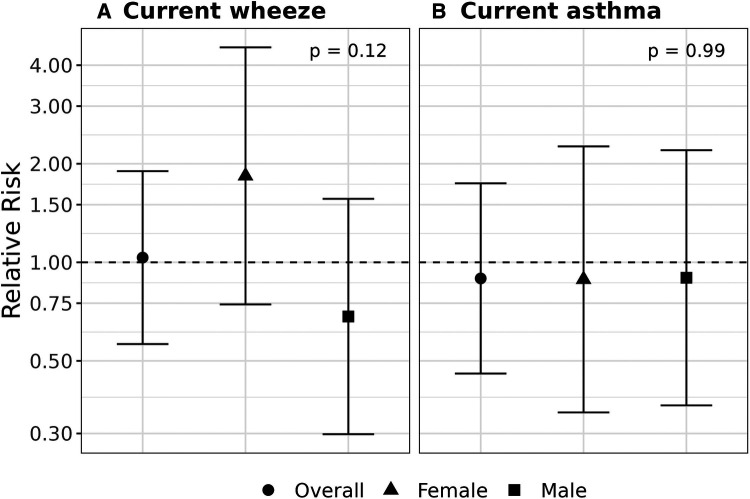
Assessment of effect modification by child sex. Relative risks (95% confidence intervals) are shown for overall, male- and female-specific associations between prenatal exposure to cleaning chemicals and development of current wheeze (**A**) and current asthma (**B**) at age 4–6 years. Models were adjusted for child age, study site, maternal race, maternal ethnicity, education at enrollment, maternal history of asthma, maternal age at delivery, maternal self-report of smoking status at enrollment, household size category, region-and inflation-adjusted household income, postnatal second-hand smoke exposure, season of birth, firstborn status, child sex, and a multiplicative interaction term for exposure to cleaning chemicals by child sex.

We did not observe statistical evidence of an interaction between prenatal exposure to cleaning chemicals and sex on development of current wheeze and current asthma. Results suggest an adverse association limited to females for the current wheeze outcome (RR 1.82, CI: 0.76, 4.37, p_interaction_ = 0.13) compared to males (RR 0.68, CI: 0.29, 1.58); however, the confidence interval was wide and included the null. Effect estimates for current asthma were less than one and did not meet statistical significance in stratified analyses by child sex (Figure 1).

The participants included in our study population were similar in all characteristics except maternal education and household income, which were both somewhat higher in the analytic sample, on average, than those excluded (Supplementary Table S1). We did not observe any statistically significant associations between our primary exposure and current wheeze or current asthma in the sensitivity analyses described above (see Supplementary Material for results). Specifically, when excluding the GAPPS 4–6 exposure recalls, we observed no significant association between the exposure and current wheeze (RR 0.94, CI: 0.50, 1.79) or current asthma (RR 0.92, CI: 0.48, 1.75) (Supplementary Table S2). However, additional adjustment for cotinine and bronchiolitis in a subpopulation in which these data elements were collected led to elevated effect estimates, though with 95% confidence intervals that included the null (RR 1.88, CI: 0.78–4.55) (Supplementary Table S1).

Additionally, upon disaggregation of the exposure classification by question, we did not observe a significant association between exposure and current wheeze (RR 0.81, CI: 0.42, 1.54) or current asthma (RR 0.88, CI: 0.42, 1.85) among mothers who cleaned floors, sinks, or toilets at their job during pregnancy. Similarly, there was not a significant association between exposure and current wheeze (RR 1.39, CI: 0.71, 2.73) or current asthma (RR 1.03, CI: 0.49, 2.15) among mothers who used janitorial chemicals or cleaners some days or every day at their job during pregnancy (Supplementary Table S3).

## 4. Discussion

This study investigated the association between occupationally related maternal exposure to cleaning chemicals during pregnancy and childhood respiratory outcomes in a combined U.S. pregnancy cohort. We found no evidence of an association between prenatal exposure to cleaning chemicals and childhood wheeze or asthma. Our findings suggested a possible sex-specific adverse association between exposure and current wheeze in females, although this result was not statistically significant.

Prior studies have investigated the link between maternal occupational exposures to cleaning products in the prenatal period and childhood respiratory outcomes. Our findings are consistent with results reported by Pape et al., who studied the association between parental occupational pre- and post-conception exposure and asthma in 3,985 offspring participating in the Respiratory Health in Northern Europe, Spain and Australia generation study (24). The authors found that parental occupational exposure to reactive chemicals, including disinfectants and cleaning chemicals, in pre- and post-conception (including the prenatal period) was not related to offspring asthma at 0–15 years of age. While maternal exposure to reactive chemicals increased the odds for early-onset asthma (0–3 years) [odds ratio (OR) 1.65, CI: 0.98, 2.77], no association was found for maternal chemical exposures and late-onset asthma (4–15 years) (OR 1.03, CI: 0.73, 1.45). The latter corresponded to a more similar child age group to our study population. Tagiyeva et al. found that maternal prenatal occupational exposure to biocides/fungicides was associated with wheeze at medium/high intensity exposure (OR 1.23, CI: 1.07, 1.40), but not with wheeze at low exposure intensity (OR 1.06, CI: 0.93, 1.20), asthma at low exposure intensity (OR 0.96, CI 0.79, 1.17) or asthma at medium/high exposure intensity (OR 1.20, CI: 0.98–1.47) in 7,088 children at 7 years of age (23). Christensen et al. found that prenatal exposure to low molecular weight (LMW) agents, identified as an exposure based on job codes for cleaners, had a borderline non-significant adverse association with asthma in 7-year-old children in the Danish National Birth Cohort (22). However, both maternal postnatal exposure to LMW agents and the combined effects of prenatal and postnatal exposure were associated with asthma. In contrast, Tjalvin et al. found that maternal occupational exposure to cleaning agents starting before conception and continuing through pregnancy were associated with childhood asthma: (OR 1.56, CI: 1.05, 2.31), childhood asthma with nasal allergies (OR 1.77, CI: 1.13, 2.77), and childhood wheeze and/or asthma (OR 1.71, CI: 1.19, 2.44) before 10 years of age among 3,318 children in two multi-national cohorts (10).

Previous studies investigating non-occupational exposures to cleaning products during pregnancy and childhood asthma and allergic disorders offer useful paradigms for comparison when evaluating our results. These studies also yielded mixed results. Bably et al. analyzed 400 children with a mean age of 6 years (SD 2.9) from Pakistan and demonstrated an association between prenatal exposure to scented cleaning products or perfume in the home with nocturnal cough among children, but not current asthma status or nocturnal symptoms of wheezing, shortness of breath, and chest tightness (40). In a study investigating household use of cleaning products during pregnancy, Casas et al. found that use of sprays or air fresheners was associated with higher prevalence of lower respiratory tract infections (LRTI) and use of sprays or solvents during pregnancy was associated with a higher prevalence of wheezing in the first year of life (41). Sherriff and colleagues reported a dose-dependent relationship between prenatal domestic use of chemical products, including disinfectants, cleaners, and fragrances, and persistent wheezing in the first 3.5 years of life, though significance differed by wheeze phenotype (42).

Biological mechanisms by which cleaning chemical exposure during the prenatal period affect respiratory health in children are not fully understood. Many cleaning products contain both irritants and sensitizers (9). The main sensitizers contained in cleaning products are disinfectants, quaternary ammonium compounds, amine compounds, and fragrances, whereas airway irritants in cleaning products include bleach, solvents, hydrochloric acid, alkaline agents, and phthalates all of which are commonly mixed together (9, 43). Many cleaning agents are LMW chemicals and are lipophilic, so transplacental diffusion may alter fetal airway development (10, 44, 45). Several human studies suggest maternal cytokines, specifically cytokines released from CD4+ Th2 T helper cells and type 2 innate lymphoid cells, mediate childhood asthma risk; however, whether this association is due to maternal cytokines passing through the placenta from maternal to fetal circulation or by modulating placental cytokine release is not clear (3). Another review found that prenatal exposure to common environmental allergens and chemicals, including tobacco smoke, organic pollutants, metals and outdoor air pollutants, may alter distributions of immune system cells, immunoglobulins and cytokine patterns in neonate cord blood (8). This derangement was postulated to result in predisposition of infants to respiratory infections during the early postnatal period and potentially an increased risk of wheeze and asthma in childhood.

Sex-dependent biological mechanisms have been implicated in asthma development (26). Prior findings have been mixed regarding effect modification by sex in the relationship between prenatal environmental exposures and child airway outcomes (27, 28) but few studies have specifically focused on prenatal occupational exposure to cleaning chemicals. Similar to our results, Christensen et al. did not find effect modification by child sex in the association between occupational exposure to LMW agents and childhood asthma (22). The prevalence of asthma is higher in boys than in girls in pre-adolescence, though the mechanism by which sex hormones regulate asthma pathophysiology is complex and requires further investigation (21, 46).

Excluding GAPPS 4–6 recall survey data and adjusting the main model for cotinine and/or bronchiolitis altered the effect estimates from less than one to greater than one. The greatest change was in the association between exposure and current asthma; after adjusting for bronchiolitis, the estimated risk ratios approached 2, though the CIs widened significantly potentially due to reduced sample size. Acute LRTI such as bronchiolitis during infancy has been found to be a strong risk factor for childhood asthma (1, 47, 48). More research on the relationship between environmental factors, including prenatal occupational cleaning product exposures, and early childhood LRTI and asthma is warranted.

Because our characterization of mothers’ cleaning chemical exposure was based on a composite measure that included job category, specific task, and frequency of chemical use, we separately examined two classifications of exposure, defined by single survey questions, as a sensitivity analysis. While the questionnaire did allow for more granular and comprehensive ascertainment of mothers’ exposure by including specific tasks and frequency rather than a single job title or category, mothers may have been misclassified as not exposed based on the wording of the questions. As in studies defining exposure through job exposure matrices, in which job titles constitute a proxy for exposure to specific agents and average exposures are often based on expert evaluation of job category, any exposure misclassification is likely to be non-differential, biasing the association towards the null (10, 24). There were very few (*N* = 10) respondents who worked as a janitor or house cleaner, suggesting that we were underpowered to investigate routine, intense occupational exposure. Furthermore, this group exhibited complete overlap with those who cleaned floors, sinks or toilets at her job, so they could not be disaggregated for separate analysis. While none of the associations reached significance, the effect estimates for those who cleaned floors, sinks or toilets at their job were less than one, whereas the effect estimates for those who used janitorial chemicals or cleaners at their jobs some days or every day were greater than one. This suggests that improved exposure classification that better approximates “dose” through frequency of use and more specific chemical data vs. more crude measures based on job duties or job type are important considerations for future research.

Our study had several strengths. Our findings contribute to a very limited and mixed literature on maternal occupational exposures, specifically during the prenatal period, and child airway health. We were able to examine the association between prenatal exposure to occupationally associated cleaning chemicals and risk of developing childhood wheeze and asthma in a U.S. based cohort comprising several cities with robust adjustment for mother and child demographic, behavioral and socioeconomic covariates and potential confounders (29).

Several limitations should be considered. Maternal occupational exposures during pregnancy were assessed retrospectively at visits with existing knowledge about whether the respiratory outcomes had occurred, which may introduce recall bias. Despite having a robust set of covariates known to influence asthma risk, we did not have data on other chemicals and products that mothers could have been exposed to associated with occupational use of cleaning chemicals or outside of work. The specific wording of the job information could include a myriad of job tasks, and occupational cleaning tasks could also confer more or less exposure to other agents, such as dust, animal dander, microbes, indoor air pollutants, all of which may impact risk of childhood asthma. Our exposures of interest in the prenatal period were highly correlated with those in the preconception and postnatal period; however, we did not have the power in this study to differentiate exposure periods to perform separate analyses. Thus, despite inclusion of numerous covariates in our models, we cannot rule out residual confounding by other factors. Furthermore, we were unable to control for application method, dose, job duration, or use of protective equipment. The sample size and number of outcomes in our final study population limited our statistical power to detect differences among exposed compared to non-exposed. The outcomes of interest were only observed among mothers who identified as White, Asian and other race and these race categories along with median income of the overall sample (>$100,000) limit generalizability of this analysis, especially given that asthma has been found to be more prevalent among Black and Hispanic children and among children living in households with lower income (49). Finally, diagnosis of asthma in children can be challenging especially in younger ages, though our study population was comprised of children at or near school age where clinical history allows more confident detection despite lack of more objective measures such as spirometry. Child airway outcome definitions may be influenced by caregiver recognition of symptoms in their children, healthcare access and utilization, and accurate recall of symptoms, medications, and diagnoses at the time of ISAAC survey administration. However, any outcome misclassification would have likely been non-differential with regards to exposure. Furthermore, symptom-based history is a broadly accepted approach to childhood asthma diagnosis and our questions were derived from the validated ISAAC questionnaire (32, 33) which remains the most widely used across the globe standardized survey to assess asthma in children (27, 28, 35, 50–54).

Childhood asthma is a chronic disease and serious public health problem that can have significant lifelong health implications. The prenatal period is a crucial period during which environmental influences, including maternal occupational exposures, can shape child respiratory health. Given the widespread use of cleaning products, amplified during the COVID epidemic, research is needed to address the role of maternal occupational exposure to specific compounds found in cleaning chemicals on offspring respiratory outcomes. Future studies should investigate more diverse and representative U.S. populations and larger sample sizes to inform our understanding. Better characterization of exposure to include ingredients of cleaning products are needed, and future studies should include more quantitative assessment of exposure, including dose, timing, and duration. Such data can better inform appropriate strategies for protecting pregnant individuals from potentially hazardous occupational exposures.

In conclusion, we did not find support for our hypothesis that maternal report of occupations using cleaning chemicals or use of cleaning chemicals at work is associated with childhood wheeze or asthma in the ECHO-PATHWAYS combined cohort, nor did we find statistically significant evidence of sex-specific associations. However, our results provide support for needed further investigation in other cohorts. Our study contributes to the emerging body of literature of prenatal occupational exposures and risk of adverse child health outcomes.

## Data Availability

The raw data supporting the conclusions of this article will be made available by the authors, without undue reservation.
