# Supplementary material for: Association between maternal occupational exposure to cleaning chemicals during pregnancy and childhood wheeze and asthma
Source: Front Epidemiol. 2023 Apr 19;3:1166174. doi: 10.3389/fepid.2023.1166174 (PMC10691794; doi:10.3389/fepid.2023.1166174)
Supplement: Supplementary file 1 [file Datasheet1.docx]

Supplementary Material

*** Correspondence:** Corresponding Author: Melissa A. Herrin, [mahury@uw.edu](mailto:mahury@uw.edu)

# Supplementary Figures and Tables


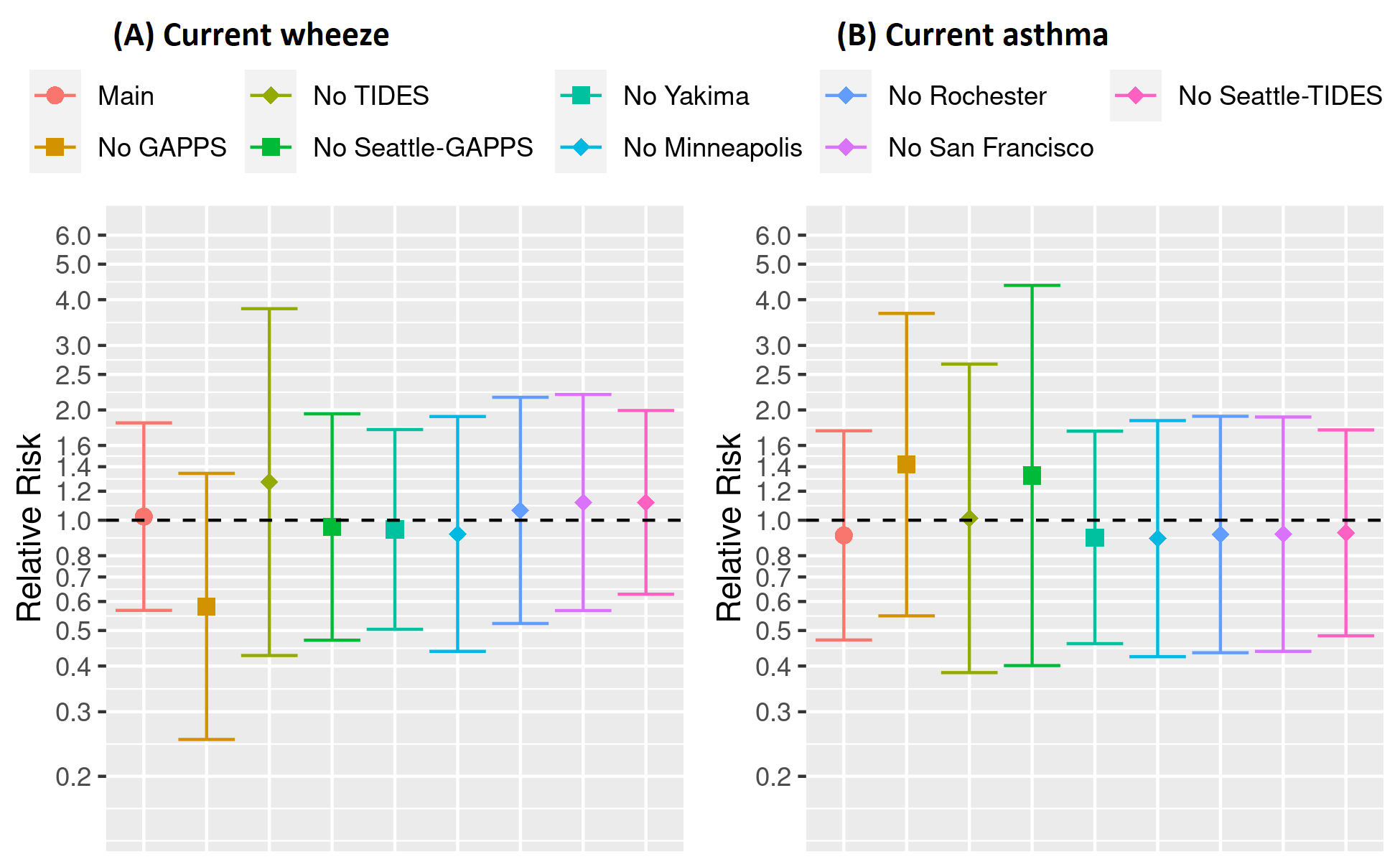
**Supplementary Figure 1.** Association between maternal occupational exposure to cleaning chemicals during pregnancy and airway outcomes leave-one-cohort or leave-one-site out analyses.

**Supplementary Table 1.** Descriptive characteristics of the study population and larger combined cohort who attended age 4-6 study visits where airway outcomes were ascertained.

|  | **Included^a^** | **Excluded** | |
| --- | --- | --- | --- |
|  | **(N=453)** | **(N=782)** | |
|  |  |  |  |
| **Maternal race** |  |  |  |
| White | 346 (76.4%) | 555 (71.0%) |  |
| Black | 20 (4.4%) | 60 (7.7%) |  |
| Asian | 24 (5.3%) | 33 (4.2%) |  |
| Native Hawaiian/Other Pacific Islander | 1 (0.2%) | 1 (0.1%) |  |
| American Indian/Alaska Native | 2 (0.4%) | 7 (0.9%) |  |
| Other | 26 (5.7%) | 39 (5.0%) |  |
| Multiple race | 16 (3.5%) | 37 (4.7%) |  |
| Missing | 18 (4.0%) | 50 (6.4%) |  |
| **Maternal ethnicity** |  |  |  |
| Hispanic or Latino | 38 (8.4%) | 92 (11.8%) |  |
| Not Hispanic or Latino | 401 (88.5%) | 654 (83.6%) |  |
| Missing | 14 (3.1%) | 36 (4.6%) |  |
| **Maternal education** |  |  |  |
| Less than high school | 14 (3.1%) | 47 (6.0%) |  |
| High school | 79 (17.4%) | 189 (24.2%) |  |
| College/technical school | 170 (37.5%) | 291 (37.2%) |  |
| Graduate or Professional degree | 190 (41.9%) | 247 (31.6%) |  |
| Missing | 0 (0%) | 8 (1.0%) |  |
| **Maternal history of asthma** |  |  |  |
| Yes | 60 (13.2%) | 100 (12.8%) |  |
| No | 365 (80.6%) | 652 (83.4%) |  |
| Missing | 28 (6.2%) | 30 (3.8%) |  |
| **Maternal Delivery Age (years)** |  |  |  |
| Mean (SD) | 32.1 (5.3) | 31.0 (3.5) |  |
| Median (IQR) | 32.0 (29.0 – 36.0) | 31.0 (27.0-35.0) |  |
| Missing | 29 (6.4%) | 27 (3.5%) |  |
| **Child sex** |  |  |  |
| Male | 246 (54.3) | 372 (47.6%) |  |
| Female | 207 (45.7%) | 410 (52.4%) |  |
| **Preterm birth** |  |  |  |
| Yes | 67 (14.8%) | 105 (13.4%) |  |
| No | 364 (80.4%) | 658 (84.1%) |  |
| Missing | 22 (4.9%) | 19 (2.4%) |  |
| **Season of birth** |  |  |  |
| Warm | 237 (52.3%) | 413 (52.8%) |  |
| Cold | 216 (47.7%) | 369 (47.2%) |  |
| **Birthweight (grams)** |  |  |  |
| Mean (SD) | 3252 (705.6) | 3295 (672.0) |  |
| Median (IQR) | 3316 (2940 - 3710) | 3354 (2963-3695) |  |
| Missing | 120 (26.5%) | 17 (2.2%) |  |
| **Firstborn status** |  |  |  |
| Yes | 130 (28.7%) | 233 (29.8%) |  |
| No | 305 (67.3%) | 542 (69.3%) |  |
| Missing | 18 (4.0%) | 7 (0.9%) |  |
| **Ever bronchiolitis** |  |  |  |
| Yes | 31 (6.8%) | 62 (7.9%) |  |
| No | 302 (66.7%) | 625 (79.9%) |  |
| Missing | 120 (26.5%) | 95 (12.1%) |  |
| **Child age at 4-6 visit (years)** |  |  |  |
| Mean (SD) | 5.8 (0.7) | 5.9 (0.7) |  |
| Median (IQR) | 6 (5.3 – 6.2) | 6.1 (5.3-6.4) |  |
| Missing | 18 (4.0%) | 0 (0%) |  |
| **Household size** |  |  |  |
| <4 | 80 (17.7%) | 146 (18.7%) |  |
| 4 | 204 (45.0%) | 290 (37.1%) |  |
| 5 | 83 (18.3%) | 143 (18.3%) |  |
| >5 | 50 (11.0%) | 77 (9.8% |  |
| Missing | 36 (7.9%) | 126 (16.1%) |  |
| **Adjusted income ($USD)** |  |  |  |
| Mean (SD) | $114,004 ($56,745) | $94,870 ($59,076) |  |
| Median (IQR) | $110,813 ($67,648 - $172,511) | $82,987 ($46,820-$141,425) |  |
| Missing | 32 (7.1%) | 122 (15.6%) |  |
| **Smoking self-report** |  |  |  |
| Yes | 15 (3.3%) | 31 (4.0%) |  |
| No | 436 (96.2%) | 737 (94.2%) |  |
| Missing | 2 (0.4%) | 14 (1.8%) |  |
| **Averaged cotinine** |  |  |  |
| Mean (SD) | 29.7 (169.2) | 35.6 (176.6) |  |
| Median (IQR) | 0.01 (0.01 – 0.06) | 0.01 (0.01-0.3) |  |
| Missing | 113 (24.9%) | 145 (18.5%) |  |
| **Postnatal second-hand smoke exposure** |  |  |  |
| Yes | 127 (28.0%) | 221 (28.3%) |  |
| No | 300 (66.2%) | 482 (61.6%) |  |
| Missing | 16 (3.5%) | 79 (10.1%) |  |
| **Cohort Site** |  |  |  |
| **GAPPS** |  |  |  |
| Seattle, WA | 164 (36.2%) | 162 (20.7%) |  |
| Yakima, WA | 75 (16.6%) | 230 (29.4%) |  |
| **TIDES** |  |  |  |
| Minneapolis, MN | 56 (12.4%) | 105 (13.4%) |  |
| Rochester, NY | 49 (10.8%) | 114 (14.6%) |  |
| San Francisco, CA | 63 (12.9%) | 87 (11.1%) |  |
| Seattle, WA | 46 (10.2%) | 84 (10.7%) |  |

^a^Participants were included if they had completed both the occupational exposure questionnaire and the International Study of Asthma and Allergies in Childhood (ISAAC) questionnaire at child age 4 – 6 years.

**Supplementary Table 2**. Association between maternal occupational exposure to cleaning chemicals during pregnancy and airway outcomes additionally adjusted for cotinine or bronchiolitis after exclusion of GAPPS age 4-6 recall surveys (N=357).

|  | **Main model^a^** | | **Main model^a^ with cotinine** | | **Main model^a^ with bronchiolitis** | | **Main model^a^ with cotinine and bronchiolitis** | |
| --- | --- | --- | --- | --- | --- | --- | --- | --- |
| Primary outcomes | Adjusted RR (95% CI) | p-value | Adjusted RR (95% CI) | p-value | Adjusted RR (95% CI) | p-value | Adjusted RR (95% CI) | p-value |
| Current wheeze | 0.94 (0.50 – 1.79) | 0.86 | 1.30 (0.54 – 3.14) | 0.56 | 1.24 (0.63 – 2.46) | 0.53 | 1.21 (0.61 – 2.41) | 0.59 |
| Current asthma | 0.92 (0.48 – 1.75) | 0.79 | 1.31 (0.54 – 3.19) | 0.55 | 1.85 (0.78 – 4.43) | 0.17 | 1.88 (0.78 – 4.55) | 0.16 |

^a^ Primary model was re-analyzed excluding the GAPPS 4-6 recall survey data

**Supplementary Table 3.** Association between maternal occupational exposure to cleaning chemicals during pregnancy and airway outcomes by classification of exposed status.

|  | **Cleaned floors, sinks, or toilets^a^** | | **Used janitorial chemicals or cleaners some days or every day^b^** | |
| --- | --- | --- | --- | --- |
| Primary outcomes | Adjusted RR (95% CI) | p-value | Adjusted RR (95% CI) | p-value |
| Current wheeze | 0.81 (0.42 – 1.54) | 0.52 | 1.39 (0.71 – 2.73) | 0.34 |
| Current asthma | 0.88 (0.42 – 1.85) | 0.74 | 1.03 (0.49 – 2.15) | 0.95 |

^a^ Association between prenatal maternal occupation exposure to cleaning chemicals and airway outcomes using the main model among those who answered “Yes” to “Did the biological mother do any of the following activities at her job during pregnancy: Clean floors, sinks, or toilets?”

^b^ Association between prenatal maternal occupation exposure to cleaning chemicals and airway outcomes using the main model among those who answered “Some days” or “Every day” to “How often did the biological mother use janitorial chemicals or cleaners during pregnancy?”

**Supplementary Table 4.** Association between maternal occupational exposure to cleaning chemicals during pregnancy and airway outcomes adjusted by child age at recall questionnaire completion visit.

|  | **Main model adjusted by child age at recall questionnaire completion visit** | |
| --- | --- | --- |
| Primary outcomes | Adjusted RR (95% CI) | p-value |
| Current wheeze | 1.03 (0.54 – 1.97) | 0.92 |
| Current asthma | 0.89 (0.43 – 1.81) | 0.74 |

**Supplementary Table 5.** Association between maternal occupational exposure to cleaning chemicals during pregnancy and airway outcomes main model without using multiple imputation by chained equations (MICE).

|  | **Main model without MICE** | |
| --- | --- | --- |
| Primary outcomes | Adjusted RR (95% CI) | p-value |
| Current wheeze | 0.87 (0.41 – 1.85) | 0.72 |
| Current asthma | 0.80 (0.36 – 1.76) | 0.58 |
